# The Impact of Various Methods of Obesity Treatment on the Quality of Life and Mental Health—A Narrative Review

**DOI:** 10.3390/ijerph20032122

**Published:** 2023-01-24

**Authors:** Marcin Hachuła, Michał Kosowski, Kaja Zielańska, Marcin Basiak, Bogusław Okopień

**Affiliations:** 1 Department of Internal Medicine and Clinical Pharmacology, Medical University of Silesia, Medyków 18, 40-752 Katowice, Poland; 2 Private Health Care Center “ALFA—MED”, Osiedle XXX-lecia 60, 44-386 Wodzisław Śląski, Poland

**Keywords:** obesity, quality of life, depression, anxiety, obesity treatment, GLP-1 analogs, dietary treatment, naltrexone/bupropion, orlistat, bariatric surgery

## Abstract

Obesity, defined as body mass index (BMI) ≥ 30 kg/m^2^, is one of the most important public health problems. Over one billion people are obese, including 650 million adults, which is 13% of the worldwide population, according to the World Health Organization (WHO). Similar to obesity, mental disorders such as depression and anxiety are huge social problems with serious health implications. There are numerous studies proving a strong link between the prevalence of obesity and depressive disorders, and being overweight is also associated with decreased health-related quality of life (HRQoL). Due to the broad negative impact of obesity on a patient’s health, proper treatment is crucial. Currently, the literature describes many methods of treatment such as dietary treatment, pharmacotherapy using glucagon-like peptide-1 (GLP-1) analogs, orlistat, naltrexone/bupropion (NB), or finally bariatric surgery. The most commonly used methods of obesity treatment significantly improve the patient’s quality of life and reduce the symptoms of depression and anxiety. The aim of our study was to summarize the knowledge about the impact of known and commonly used methods of obesity treatment (e.g., dietary treatment, bariatric surgery, and pharmacological treatment) on mental health and quality of life. For this purpose, we will try to review the current scientific data, originating from international reports.

## 1. Introduction

Obesity, defined as body mass index (BMI) ≥ 30 kg/m^2^, is without a doubt one of the most important public health issues. According to recent World Health Organization (WHO) data, over one billion people worldwide are obese, including 650 million adults, which is 13% of the worldwide population. The number has nearly tripled since 1975 and is continuously, rapidly increasing—the WHO estimates that by 2025, approximately 167 million people will become less healthy because they are overweight or obese.

The problem will become more blazing as obesity pertains also to approximately 340 million adolescents and 39 million children [1]. The impact of excessive BMI in children is especially striking, as being overweight during the first 10 years of life carries a higher risk of becoming an obese adult than having one or both obese parents [2].

For the first time in human history, the number of overweight people has exceeded the number of underweight people [3]. Previously associated with high-income Western countries, obesity is becoming a growing problem in developing countries such as China and India and among low-income populations [4,5,6]. Experience from the foregoing studies may not be appropriate for those new populations.

The connection between excessive body mass and the risk of chronic diseases is widely known and well documented. 

The best known is the relation between obesity and cardiovascular diseases [7,8] such as hypertension, coronary heart disease, and chronic heart failure, as well as arrhythmias and both ischemic and hemorrhagic stroke [9,10]. Nearly 70% of obese patients’ deaths are due to cardiovascular disease [10]. BMI over 25 is the most important risk factor for insulin resistance [11] and diabetes mellitus. The combined health effects of obesity and diabetes mellitus are so great that the term “diabesity” was introduced [8]. Global Burden of Disease Study 2015 (GBD 2015) Obesity Collaborators found convincing evidence connecting obesity with a number of malignancies: esophageal cancer, colon and rectum cancer, liver cancer, gallbladder and biliary tract cancer, pancreatic cancer, breast cancer, uterine cancer, ovarian cancer, kidney cancer, thyroid cancer, and leukemia [10].

In recent years of the COVID-19 pandemic, obesity has become a more prevalent problem, as it has been reported that the risks of hospitalization, intensive care unit admission, invasive mechanical ventilation, and death are higher with an increasing BMI [12,13].

As such, obesity becomes a center of interest for a large part of the medical community, including professionals from various fields.

Similar to obesity, the problem of depression and anxiety disorders is a very important problem on a global scale, with serious social implications [10,14]. The group of depression and anxiety disorders is one of the most common types of mental disorders [15]. They cause significant impairment in patients’ everyday lives [16]. It also increases patients’ aversion to using health services, which negatively affects the control of chronic diseases [17].

A very important issue seems to be the fact that numerous meta-analyses indicate a strong relationship between the occurrence of obesity and depressive disorders [18,19,20]. Moreover, four longitudinal meta-analyses have shown that this relationship is bidirectional, i.e., obesity and depressive disorders increase the risk of depression, and vice versa, the diagnosis of depressive disorders in a patient’s history increases the risk of obesity [21,22,23,24]. The relationship between the occurrence of depression and obesity is much more noticeable in the case of abdominal obesity than in the case of other types of this disorder, which was shown by the meta-analysis conducted by Xu et al. [19]. This is probably due to the most frequent pathomechanism that links these two disease entities, which is excessive stimulation of the hypothalamic–pituitary–adrenal (HPA) axis, and this leads to a chronic increase in cortisol level, which is very important in the pathogenesis of both depression and abdominal obesity [25,26]. However, the activation of the HPA axis is not the only factor postulated by scientists to link depression and obesity. Other very important factors include diet habits, intestinal microbiome disorders, the inflammatory process in the body, and finally the use of antidepressants [27].

For many years, scientists have also been looking for a similar relationship in other psychiatric disorders, including anxiety disorders. Numerous studies have shown a relationship between the occurrence of anxiety disorders and obesity [28,29], and a meta-analysis conducted in 2010 also proved that this relationship occurs regardless of gender and that the prevalence of obesity has also been associated with anxiety disorders in the past in the examined patients [30].

The impact of obesity is not only limited to health issues but also to quality of life (QoL), as it increases the likelihood of death and lowers QoL indicators that are characterized by social interaction, low self-esteem, social isolation, stress, and mental illnesses [31]. 

Quality of life is defined as an individual’s own assessment of well-being. In research, the quantification of QoL related to health status is referred to as health-related QoL (HRQoL)—“a multidomain concept that represents the patient’s general perception of the impact of an illness and its treatment on physical, psychological, and social aspects of life” [32]. Tools designed to evaluate HRQoL in reviewed studies can be divided into three groups: generic (SF-36, RAND 36), obesity-specific (Moorehead-Ardelt Quality of Life, IWQOL-Lite), and other (ADDQoL and GIQLI).

According to Kolotkin et al.’s review, obesity is associated with decreased HRQoL, particularly in those with class III obesity (BMI ≥ 40 kg m^−2^). Moreover, the physical aspects of HRQoL seem to be more closely associated with the degree of obesity than the mental aspects of HRQoL [33].

Due to the wide negative impact of obesity on the health of patients, its appropriate treatment is very important. Currently, the literature describes many methods of treating this disease, such as dietary treatment, pharmacotherapy using glucagon-like peptide-1 (GLP-1) analogs, orlistat, or naltrexone/bupropion (NB), or finally bariatric surgery.

The aim of our study was to summarize the knowledge about the impact of known and commonly used methods of obesity treatment on the mental health of patients and their quality of life. For this purpose, we will try to systematically review the current scientific data originating from international reports.

## 2. Materials and Methods

In this publication, we aimed to review the current scientific data from international reports. Two independent authors searched the “PubMed” and “Embase” databases to select proper articles. Databases were screened in order to isolate reports according to the following key phrases: “obesity and quality of life”, “obesity and depression”, obesity and anxiety”, “obesity and affective disorders”, “dietary treatment quality of life”, “dietary treatment depression”, “dietary treatment anxiety”, “dietary treatment affective disorders”, “GLP-1 analogues quality of life”, “GLP-1 analogues depression”, “GLP-1 analogues anxiety”, “GLP-1 analogues affective disorders”, “orlistat quality of life”, “orlistat depression”, “orlistat anxiety”, “orlistat affective disorders”, “naltrexone/bupropion quality of life”, “naltrexone/bupropion depression”, “naltrexone/bupropion anxiety”, “naltrexone/bupropion affective disorders”, “bariatric surgery quality of life”, “bariatric surgery depression”, “bariatric surgery anxiety” and “bariatric surgery affective disorders”. Manuscripts published from 1990–2022 were taken into consideration. Non-English articles were excluded from the analysis. Apart from the review methodology, the authors have also included other articles necessary to explain the mechanisms and methods of obesity treatment.

## 3. Impact of Non-Pharmacological Obesity Treatment on Mental Health

The most valuable non-pharmacological methods in obesity treatment are physical activity and dietary treatment. Despite recently intensively developed modern methods of treating obesity, the basic form of therapy in patients is still a change in diet habits. Pharmacological treatment (for example, GLP-1 analogs) or bariatric surgery is only a form of additional therapy aimed at enhancing non-pharmacological forms of therapy [34]. Dietary treatment mainly uses three types of diets: low-fat/low-energy diets; Mediterranean/low-energy diets; low-carb/non-energy-reduced diets, whose clinical effects in comparative studies do not differ significantly from each other [35]. It should be remembered, however, that dietary treatment, apart from its beneficial effect on the reduction of weight and the accompanying complications, such as cardiovascular incidents, also affects the mood of patients. As in the case of the relationship between obesity and mental disorders, the relationship with diet seems to be bidirectional. This means that a properly selected diet can improve the symptoms of mental disorders, and these disorders can affect the diet itself [36]. The literature describes many mechanisms explaining this relationship. One of them is the direct effect of diet on the functioning of the central nervous system, as in the case of a high-fat diet, which is believed to be the cause of mood disorders due to the influence of fats on serotonergic transmission [37,38]. Another mechanism presented by researchers is the influence of diet on the intestinal microbiota, whose abnormalities may also cause mood disorders [39]. In reference to the mechanisms described above, numerous clinical studies have been conducted to determine the impact of particular types of diets on the progression and treatment of certain mental illnesses.

The first studies conducted on the population of patients with undiagnosed depression gave very diverse results [40]. On the one hand, they did not show significant differences in the severity of depressive symptoms in people on a low-fat diet versus the control group [41]. On the other hand, they showed a reduction in depressive symptoms after dietary treatment in such groups of patients as the elderly or patients suffering from breast cancer [42,43]. Moreover, many other studies have shown that non-pharmacological methods of obesity treatment in the form of dietary intervention and physical activity, although they did not directly affect the severity of depressive symptoms, significantly improved the quality of life of patients [41,42,43,44,45]. In a large randomized clinical trial conducted by Assaf AR et al., it has been proven that the use of a low-fat diet statistically significantly improves the quality of life of patients measured using the RAND 36-Item Health Survey form. The improvement was visible in three HRQoL subclasses: general health, physical functioning, and vitality. Moreover, the authors of the study pointed out that the dietary intervention improved the quality of life in a manner independent of weight loss [41]. Another clinical study showed that dietary intervention alone improved the quality of life of patients, regardless of whether it was accompanied by physical activity or not. This suggests that a properly balanced diet improves patients’ quality of life and mental health not only by lowering calories and thus body weight but also by the direct impact of nutrients on patients’ psyches [44]. Similar observations were also made in a group of patients with undiagnosed depression who followed a Mediterranean diet [46]. The issue of dietary treatment looks completely different in a group of patients who have been diagnosed with depressive disorders. One of the most important studies on this issue is the study Supporting the Modification of Lifestyle in Lowered Emotional States (SMILES), in which it was proven that the use of the Mediterranean diet in patients with confirmed depression reduces depressive symptoms compared to the group of patients who used a standard diet [47]. Moreover, this type of diet, as evidenced by numerous clinical studies, has a positive effect on the quality of patients’ sleep, prolonging its duration and reducing the risk of insomnia, which translates into a significant improvement in the quality of life [48,49,50]. Two studies were conducted on a group of patients with symptoms of depression and exposure to mental stress, in which the impact of dietary education on symptom reduction was examined, and although the first of these studies did not show a statistically significant improvement, the second proved that dietary interventions can improve the mental state of these patients [51,52]. To sum up, although the evidence for the existence of a relationship between dietary treatment and the reduction of depressive symptoms is not clear, such an effect is visible in the group of patients diagnosed with depression, so additional clinical trials are needed to solve this problem unequivocally. 

A much less studied yet still unexplained problem is the impact of dietary treatment on disorders that are as common as depression, i.e., anxiety disorders. Although the mechanism linking obesity and anxiety disorder is consistent with the one linking obesity with depression, clinical trials examining the effect of dietary treatment on the intensity of anxiety in patients do not indicate a significant reduction after the use of this type of therapy [40]. However, it should be noted that in all of these studies, the initial level of anxiety in the included group of patients was relatively low, which could have distorted these outcomes. Especially since one clinical trial in patients with affective disorders found a statistically significant reduction in anxiety levels [47]. For this reason, it seems important to conduct additional randomized clinical trials in the future, which will allow us to assess the effect of dietary treatment on anxiety disorders.

At the end of the dietary topic, we would like to briefly summarize the newest knowledge about dietary treatment. The data showed that the main strategy for weight management should be focused on reducing the amount of calories in consumed meals [53]. Clinical trials, e.g., Preventing Overweight Using Novel Dietary Strategies (POUNDS LOST), proved that weight loss was similar in different types of diets. The analysis confirmed that the amount of calories played a key role in weight reduction [54]. In the large multicentered PREMIER trial, researchers examined the impact of eating patterns, e.g., the DASH diet, on weight loss. The results showed no differences after 6 months [55]. Low-energy-dense nutrition could help reduce energy intake by enhancing satiety [56]. The impact of various types of diets on weight reduction is described in detail in a review prepared by Smethers et al. [53]. 

Physical activity is an essential factor contributing to both physical and mental well-being [57]. The correlation between regular exercise and better quality of life, life satisfaction, and happiness has been reported in all age groups [58,59]. The positive impact of physical activity on mental health is relatively well understood and documented [60,61]. 

The positive effect on mental health is not limited only to patients suffering from psychological disorders but also applies to healthy individuals. Some sources suggest that regular physical activity may prevent the development of depressive disorders, but this has not been proven experimentally [57]. Physical activity is not only considered as a prevention but also is a crucial part of the treatment. In obesity therapy, it is both a form and one of the pillars of the treatment, as well as a remedial factor and a guarantee of maintaining the effects [62]. Physical exercises can be divided into two types: aerobic and anaerobic. The first focuses on weight loss, and the second one concentrates on strengthening muscles. While both of them are important in obesity treatment and have been shown to have positive effects on depressive symptoms, in the case of anxiety disorders, the results are still inconclusive. [58,59] 

## 4. The Effect of Obesity Treatment with a Combination Therapy of Naltrexone and Bupropion on Depression and Quality of Life

A very interesting issue related to the effect of pharmacotherapy used in the treatment of obesity on depressive symptoms and the quality of life of patients is the use of combination therapy (NB). This drug has been registered for the treatment of obesity since 2014. The main mechanism of action of this drug consists of proopiomelanocortin-expressing (POMC) neurons stimulation by bupropion to release melanotropin-α (α-MSH), which is responsible for melanocortin 4 receptors (MC4-R) stimulation. At the same time, POMC neurons release β-endorphin, which is an agonist of the µ-opioid receptor. The binding of β-endorphin to the µ-opioid receptors on POMC neurons is the closing point of the negative feedback loop on POMC neurons and leads to a decrease in the release of α-MSH. It has been hypothesized that this negative feedback loop blockage by naltrexone facilitates stronger and longer-lasting activation of POMC neurons and thus amplifies the effect of bupropion on energy balance [63]. Numerous clinical trials have confirmed the effectiveness of this preparation in the treatment of obesity, but also its high safety profile [64,65,66,67].

Due to the fact that obesity significantly affects the quality of life, the impact of various obesity therapies has been thoroughly investigated. In addition to the previously mentioned clinical trials, which, apart from confirming the effectiveness of obesity treatment, also confirmed the impact of NB therapy on improving the quality of life [64,66,67], there was also a study conducted by Halseth A. et al. In this randomized, multi-center clinical trial, it was proven that NB therapy significantly improves the quality of life compared to non-pharmacological methods of treating this disease [68].

Another important issue seems to be the effect of NB therapy on depressive symptoms in patients, especially since one of the components of this therapy, i.e., bupropion, is a drug registered by the Food and Drug Administration (FDA) for the treatment of major depressive disorders [69]. According to our best knowledge, studies directly describing such an impact are, at this point, still rare in the literature. However, one of the meta-analyses of five clinical trials using NB that described the side effects of the therapy is noteworthy. In this meta-analysis, attention was drawn to the fact that depressive symptoms occurred much less frequently in the NB-using group than in the placebo group, which may suggest a positive effect of such a therapy [70]. Due to these reports, it seems reasonable to hypothesize that NB has a beneficial effect on the symptoms of depression not only through weight reduction itself but also through another mechanism. However, additional research is needed to clearly prove this effect.

## 5. Influence on Mental Health by Obesity Treatment with Therapy of Orlistat

Orlistat is a medicine whose anti-obesity effects are widely known. Its primary function is to decrease intestinal fat absorption by up to 30%, it acts as a reversible inhibitor of pancreatic and gastric lipases, thereby reducing daily caloric intake [71]. Orlistat has been shown in numerous scientific studies to be effective for significant weight loss [72,73].

The link between orlistat treatment and its influence on mental disorders seems intriguing because orlistat has anxiety listed as one of the side effects [74]; however, no scientific studies confirm this information. Benazzi described a case study, where orlistat induced depression, but only one survey about this topic is available. Furthermore, a reported case involved a woman with bipolar II disorder who suffered from three episodes of depression before the therapy with orlistat began [75].

Some analysis suggests body weight reduction during therapy with orlistat improves the quality of life and reduces the intensity of depression symptoms. In patients with binge eating disorder (BED), the available data indicate that up to 65% meet the criteria for anxiety disorders and 46% for mood disorders [76]. Carlos, M. et al. found in their study that in patients with BED, orlistat therapy connected with behavioral psychotherapy and, at the same time, statistically reduced the severity of depression compared to the control group [77]. Moreover, Kiortsis observed an essential improvement in the emotional condition after weight loss with orlistat treatment in overweight women [78]. Nowadays, we have more effective medications for obesity, so the use of orlistat has decreased. However, it is still an efficient drug that could improve the quality of life for obese people.

## 6. Influence on Mental Health by Obesity Treatment with Therapy of GLP1 Analogs

Liraglutide, semaglutide, exenatide, and dulaglutide belong to the group of human GLP-1 analogs. They are a new class of glucose-lowering medications whose action on incretin mimetic activation has been approved for the treatment of type 2 diabetes mellitus, and some of them have also been approved for the treatment of obesity [79,80]. GLP-1 receptors can be found in a variety of body systems, including the central nervous system [81]. In the available medical literature, there are numerous reports on the impact of mental disorders such as depression [81]. Grant, et al, in a not-randomized trial, investigated the influence of exenatide on psychological and quality of life changes in participants diagnosed with type 2 diabetes. Treatment with GLP-1 receptor agonists resulted in a reduction in the depression scale [82]. In the cohort study of 1735 patients with newly diagnosed type 2 diabetes who had incretin-based therapies, a significant reduction in depressive symptoms was observed compared to the control group [83].

Multiple studies prove the beneficial effect of GLP-1 analog therapy on the quality of life measured with various scales. The study conducted by Kahal, et al, in young women with PCOS and obesity treated with liraglutide resulted in a significant improvement in their QoL. However, no differences were found in the risk for depression [84]. The SUSTAIN 6 was a 2-year, randomized, double-blind, placebo-controlled trial with 3297 participants that showed improvement in HRQoL in patients who were treated with semaglutide [85]. The results of an analysis of overweight or obese people with comorbidities revealed that treatment with liraglutide is associated with better health-related quality of life after one year of therapy compared to a placebo, and that this effect is stable for long observation up to three years [86,87]. The analysis of data from 4725 participants across three randomized, placebo-controlled, double-blind trials that evaluated the efficacy and safety of liraglutide proved improvements in metabolic parameters and health-related quality of life. It also showed that liraglutide’s effectiveness is caused by weight loss and by independent mechanisms not related to weight loss [88]. Chao, et al., compared the effects of intensive behavioral therapy (IBT) with IBT with liraglutide on quality of life and weight-related quality of life in an obese patient. The results showed that the addition of liraglutide to IBT appears to improve aspects of both general HRQoL and weight-related QoL [89]. In the study conducted by Lundershausen, et al, liraglutide improved certain components of the diabetes-dependent quality of life (ADDQoL) questionnaire in patients with diabetes [90]. Moreover, in the AWARD clinical trial program, dulaglutide, a GLP-1 receptor agonist, significantly improved the impact of weight on self-perception compared with insulin glargine [91]. Numerous clinical trials have shown that once-a-week GLP-1 administration, such as semaglutide and dulaglutide, appears to offer more benefits in HRQoL compared to oral medications, insulin, and daily injections of GLP-1 analogs [92]. 

There are strong indications that the mechanism of action of GLP-1 analogs in the context of reducing the severity of depressive symptoms and improving widely understood mental health and quality of life is multifaceted. Firstly, there is scientific evidence that weight loss improves mental health and reduces depression symptoms [88,93]. On the other hand, numerous pro-inflammatory cytokines such as interleukin-1, interleukin-6, and tumor necrosis factor α, that generate neuroinflammation, are involved in the pathophysiology of mental disorders such as depression and influence their severity and deterioration [94,95,96]. Chronic inflammation in the brain promotes the destruction of the serotoninergic, dopaminergic, and noradrenergic pathways [97]. Moreover, proinflammatory cytokines involved in the mechanism of depression are linked to neuronal atrophy and attenuation of neurogenesis, principally in the hippocampus region [98]. The effect of GLP-1 analogs on the reduction of pro-inflammatory cytokines has been proven in experimental animal models [99,100]. Weina, et al, demonstrated in mice that liraglutide reduces depressive behavior by increasing hippocampal neural plasticity [101].

To sum up, the anti-diabetic drugs belonging to the GLP-1 analog class have the potential to act as a therapeutic treatment for mental disorders such as depression, especially in patients with obesity or diabetes, which are strongly related to each other [102].

## 7. The Effect of Obesity Treatment with Bariatric Surgery on Depression, Anxiety and Quality of Life of Patients

The first bariatric surgery was jejunoileal bypass (JIB), performed in 1954 by A.J. Kremen [103]. Since then, bariatric surgery has become the most effective and enduring method of obesity treatment [104]. There are a number of methods currently used, such as: Roux-en-Y gastric bypass, sleeve gastrectomy, biliopancreatic diversion with duodenal switch, and implantation of devices (adjustable gastric banding, intermittent vagal blockade, gastrointestinal endoscopic devices) [105]. These methods differ in their indications and final results of weight loss, but there are a growing number of studies focused on their impact on quality of life.

HRQoL assessment tools most commonly used in the surgical literature are the Short Form-36 (SF-36) [106], Moorehead-Ardelt Quality of Life [107], Gastrointestinal Quality of Life (GIQLI) [108], and Impact of Weight on Quality of Life (IWQOL) [109]. Bariatric Analysis and Reporting Outcome System (BAROS) [110] used in many studies is not a purely HRQOL measure as it incorporates some physician-reported outcomes such as the presence of complications [111]. 

It should be noted at this point that patients qualified for bariatric obesity treatment initially have a lower quality of life compared to the population norm [112]. Małczak, et al., reviewed 47 studies involving 26,629 patients and 11 different types of surgeries. They concluded that bariatric interventions result in better HRQoL than non-surgical interventions. Moreover, HRQoL improvement is significantly greater after sleeve gastrectomy and Roux-en-Y gastric bypass compared with lifestyle intervention [113]. Sierżanowicz et al. compared 18 published studies on the long-term outcomes of bariatric surgery. They found that HRQOL after bariatric surgery tends to improve in the first 1–2 years after surgery and then deteriorate again in the first 5–6 years. However, HRQOL after 9–12 years is generally not significantly different from that observed after the first five years. Despite the decrease in HRQOL scores, in most of the reviewed studies, the value after 9–12 years was still significantly higher than at the beginning of the study, suggesting that bariatric treatment has a lasting beneficial effect on quality of life. They also demonstrated that the degree of HRQOL improvement was proportional to the level of weight reduction [114]. 

Depression and anxiety disorders in the context of bariatric surgery are much less covered in the literature. Some research suggests that mental disorders may be more common among patients undergoing bariatric surgery—a meta-analysis by Dawes et al. estimated that 23% of patients reported a current mood disorder, most commonly depression. Prevalence is higher than published rates for the general US population. The same analysis concludes that the prevalence of depression as well as the frequency and severity of depressive symptoms improve after bariatric intervention, at least in the span of the first 3 years after surgery. Evidence regarding the association between preoperative mental health conditions and postoperative weight loss is still inconsistent [115].

There are not many studies yet that have looked at how bariatric surgery affects anxiety disorders, but the evidence we have so far shows that bariatric surgery is linked to long-term improvements in both anxiety and depression [116].

## 8. Conclusions

Obesity is a civilizational disease associated with a higher risk of depression and anxiety, as well as a reduction in the quality of life of patients suffering from it. The most commonly used methods of obesity treatment, such as dietary treatment, pharmacotherapy, and surgical treatment, significantly improve the quality of life of patients and reduce the symptoms of depression and anxiety, which probably result not only from the fact of weight reduction, as in the case of GLP-1 analogs. However, there is a severe lack of unequivocal studies describing the impact and mechanism by which various types of obesity therapy affect the quality of life of patients as well as their symptoms of depression and anxiety.

## Data Availability

Not applicable.
